# LINC00899 suppresses the progression of triple-negative breast cancer via the miRNA-425/PTEN axis and is a biomarker for neoadjuvant chemotherapy efficacy

**DOI:** 10.7150/jca.100619

**Published:** 2025-02-10

**Authors:** Lianjie Niu, Yongtao Bai, Meng Yu, Xianfu Sun

**Affiliations:** 1 Department of Breast Disease, Henan Breast Cancer Center, The Affiliated Cancer Hospital of Zhengzhou University and Henan Cancer Hospital, Zhengzhou, Henan 450008, P.R. China.; 2 Department of Pharmacy, The Affiliated Cancer Hospital of Zhengzhou University and Henan Cancer Hospital, Zhengzhou, Henan 450008, P.R. China.; 3 Clinical Research Center, The Affiliated Cancer Hospital of Zhengzhou University and Henan Cancer Hospital, Zhengzhou, Henan 450008, P.R. China.; 4 Department of General Surgery, The Affiliated Cancer Hospital of Zhengzhou University and Henan Cancer Hospital, Zhengzhou, Henan 450008, P.R. China.

**Keywords:** exosome, LINC00899, microRNA-425, phosphatase and tensin homolog, triple-negative breast cancer, progression

## Abstract

**Background:** Close clinical attention has been paid to triple-negative breast cancer (TNBC) due to its poor prognosis, high recurrence and mortality rates and rapid invasion and metastasis. The present study aimed to explore the potential mechanism of LINC00899 in the progression of TNBC and its effect on the proliferation and migration of TNBC cells via the microRNA (miR)-425/phosphatase and tensin homolog (PTEN) axis.

**Methods:** For this purpose, plasma exosomes and related clinical data from 119 patients with breast cancer receiving neoadjuvant chemotherapy (59 patients with TNBC, 32 with HER2^+^ and with 28 luminal-type) and 20 healthy women were collected. Functional assays were then used to verify the role of the LINC00899/miR-425/PTEN axis in the proliferation and migration of TNBC cells.

**Results:** The results showed that the expression of LINC00899 was reduced in plasma exosomes and breast cancer cell lines, which was associated with the Ki-67 index, tumor size and the presence or absence of lymph node metastasis but was not associated with patient age, androgen receptor expression or cholangiocarcinoma thrombus. The receiver operating characteristic curve results showed that LINC00899 had a high predictive value for the pathological outcome of patients with TNBC receiving neoadjuvant treatment. The results of the functional experiments also showed that LINC00899 targeted and regulated miR-425 in TNBC, and miR-425 negatively regulated the expression of PTEN.

**Conclusions:** In conclusion, the results of the present indicated that LINC00899 may predict neoadjuvant chemotherapy efficacy in patients with TNBC and that LINC00899 inhibited the proliferation and migration of MDA-MB-231 cells via the miR-425/PTEN axis.

## 1. Introduction

Breast cancer is one of the most common malignant tumors in women worldwide 1. Triple-negative breast cancer (TNBC) accounts for approximately one-fifth of breast cancer cases, and close clinical attention has been paid to TNBC due to its poor prognosis, high recurrence and mortality rates and rapid invasion and metastasis. Early TNBC is most likely to recur within 3 years of initial treatment, and most patients die within 5 years of initial treatment. The median distant metastasis time is 25 months and the median postrecurrence survival time is 17 months, compared with other types of breast cancer 2-4.

Long non-coding RNAs (lncRNAs) are RNA molecules >200 nucleotides in length with no protein coding ability, which are widely involved in the pathophysiological regulation of malignant tumors such as liver, lung, bowel and breast cancer 5. LINC00899 is a lncRNA with a tumor suppressive effect that is located on chromosome 22q13.31. A study by Wang *et al* 6 demonstrated that the LINC00899 may serve as a novel serum biomarker for predicting the diagnosis and prognosis of acute myeloid leukemia. Furthermore, Dong *et al* 7 showed that LINC00899 promotes the proliferation and apoptosis of acute myeloid leukemia cells by regulating the microRNA(miR)-744-3p/Yin Yang 1 signaling axis. A study by Zhou *et al* [8]also showed that LINC00899 can significantly inhibit the proliferation, migration and invasion of breast cancer cells by enhancing DICER1 expression.

microRNAs (miRNAs) are a type of small non-coding RNA with only 18-25 nucleotides. miRNAs participate in various crucial cellular processes, such as changes in the cell cycle, invasion and apoptosis. A number of studies have also shown that miRNAs have an important role in numerous biological processes in breast cancer, and the imbalance of miRNA expression may lead to the malignant progression of this disease 9-11. It has been reported that superoxide dismutase 1-high fibroblast-derived exosomal miR-3960 promotes cisplatin resistance in TNBC by inhibiting BR serine/threonine kinase 2-mediated PICALM interacting mitotic regulator phosphorylation 12. Recent research has found that abnormal expression of miR-425 is associated with various cancer types 13,14. Furthermore, it has been reported that miR-425-5p and miR-451 may serve as useful biomarkers for assessing cardiovascular disease risk in patients with rheumatoid arthritis^15^. miR-425 delivered by extracellular vesicle from A549-R cells promotes the progression of non-small cell lung cancer by regulating the phosphatidylinositol-3-hydroxykinase (PI3K)/protein kinase B (AKT) signaling pathway mediated by death associated protein kinase 1^16^. In addition, miR-425-5p regulates chemotherapy resistance in colorectal cancer cells by modulating the expression levels of programmed cell death 10 both *in vivo* and *in vitro*; the expression level of miR-425-3p in liver biopsy tissue may also assist the stratification of patients with advanced hepatocellular carcinoma and guide sorafenib treatment 17.

In a previous study, miRNA microarray analysis by our research group revealed 34 miRNAs that were differentially upregulated between the MD-MB-231 TNBC cell line and the HBL-10 breast epithelial cell line 18. These 34 miRNAs were predicted to interact with long intergenic non-coding RNA, and miR-425 was identified as a potentially significant target for LINC00899 using miRcode and RNA22. The present study explored the underlying mechanism of LINC00899 in the progression of TNBC and found that LINC00899 expression was reduced in plasma exosomes and breast cancer cell lines and that LINC00899 promoted the proliferation and migration of TNBC cells by regulating the expression of miR-425/phosphatase and tensin homolog (PTEN).

## 2.Materials and Methods

### 2.1 Plasma sampling and breast cancer tissue

Peripheral blood (from 20 healthy volunteers and 119 patients with breast cancer) and breast cancer tissue (from 119 patients with breast cancer) were collected from each participant and transferred to test tubes. The test tubes containing peripheral blood were centrifuged at 1,200 x g for 15 min at 26˚C and the plasma was collected. The plasma and breast cancer tissue samples were stored in liquid nitrogen until further use. Inclusion criteria: Age: 18-70 years old (including 18 years old, 70 years old) patients with clinically pathologically confirmed breast cancer to be treated with neoadjuvant therapy, except for inflammatory breast cancer; Have clinically measurable lesions: measurable lesions shown by ultrasound, mammography, or MR (optional) within 1 month prior to randomization. Exclusion Criteria: Evidence of metastatic breast cancer (CT scan of the chest and abdomen and bone at any time point prior to diagnosis to randomization is required to rule out metastatic breast cancer; PET/CT scan as an alternative imaging modality); For this disease, chemotherapy, endocrine therapy, targeted therapy, radiation therapy, etc. have been received; The patient has a second primary malignancy, except for adequately treated skin cancer. All procedures for healthy volunteers and patients with breast cancer involved in the present study were in-line with the Declaration of Helsinki revised in 2013. The present study was approved by the Institutional Review Committee of Henan Cancer Hospital Affiliated to Zhengzhou University (Zhengzhou, China; approval no. 2022-KY-001). All participants signed a written informed consent form before recruitment.

### 2.2 Cell culture

The human breast cancer cell lines (MDA-MB-231, SKBR3, MCF-7 and MCF-10A) used in the present study were purchased from the Institute of Biochemistry and Cell Biology of the Chinese Academy of Sciences. All cells were authenticated by short tandem repeat analysis and all cell experiments were completed within 6 months. The cell incubator conditions were 5.0% CO_2_ and 37.0˚C.

### 2.3 Cell proliferation

The MDA-MB-231 cells were seeded into a 96-well plate at a density of 5x10^3^ cells/well and treated according to the experimental groups. Then, Cell Counting Kit-8 (CCK-8) reagent (10 μl) was added to the various groups. The absorbance at 450 nm was subsequently measured using an enzyme immunoassay analyzer (Thermo Fisher Scientific, Inc.). MDA-MB-231 cells were seeded at a density of 5x10^3^ cells/well into a 6-well plate, then the colonies were counted until the lesion was obvious.

### 2.4 Transwell assays

Each group of MDA-MB-231 cells transfected in serum-free medium was cultivated then added to the upper chamber of a Transwell plate. Then, 10% FBS medium was added to the lower chamber as a chemical inducer. After 48 h of incubation, MDA-MB-231 cells at the bottom of the membrane were stained with 0.1% crystal violet (Beyotime Institute of Biotechnology), and Transwell cell migration images in three randomly selected fields of view were obtained using an inverted microscope.

### 2.5 Exosome extraction and identification

The extraction and identification of exosomes using transmission electron microscopy (TEM) were as described previously 19.

### 2.6 Western blotting analysis of PTEN

The proteins extracted from each group of MDA-MB-231 cells were separated using a 10% gel (the same concentration of protein was added per group), then transferred to a membrane. The detection membrane was incubated with a PTEN antibody (1:200; Wan Lei Bio) at 4˚C for 12 h, then incubated with a secondary antibody for 1 h. For protein band visualization, the membrane was treated with enhanced chemiluminescence reagents (MilliporeSigma) and observed using the FluorChem FC2 Imaging System (ProteinSimple).

### 2.8 Reverse transcription-quantitative polymerase chain reaction (RT-qPCR)

The total RNA from exosomes or cells was extracted according to the steps of the reagent kit. RT-qPCR was performed according to the operating procedures of the SYBR Premix Dimer Eraser kit (Takara Bio, Inc.) using the LightCycler 480 II detection system (Roche Diagnostics). The primer sequences are listed in Table 1. The relative expression levels of LINC00899, miR-425 and PTEN were quantified using the expression levels of U6 and GAPDH as a reference. The relative gene expression levels were calculated using the 2^-ΔΔCq^ method.

### 2.9 Statistical analysis

SPSS version 24.0 (IBM, Corp.) was used for the statistical analysis of data, and all results are presented as the mean ± standard deviation. The comparison of two groups was conducted using the unpaired t-test. Correlation analysis was conducted using the Pearson analysis method. Receiver operating characteristic (ROC) curve of subjects was used to determine the accuracy of the prediction of neoadjuvant chemotherapy efficacy in patients with breast cancer. P<0.05 was considered to indicate a statistically significant difference.

## 3. Results

### 3.1 LINC00899 targets and regulates miR-425 in TNBC

**Figure 1:**
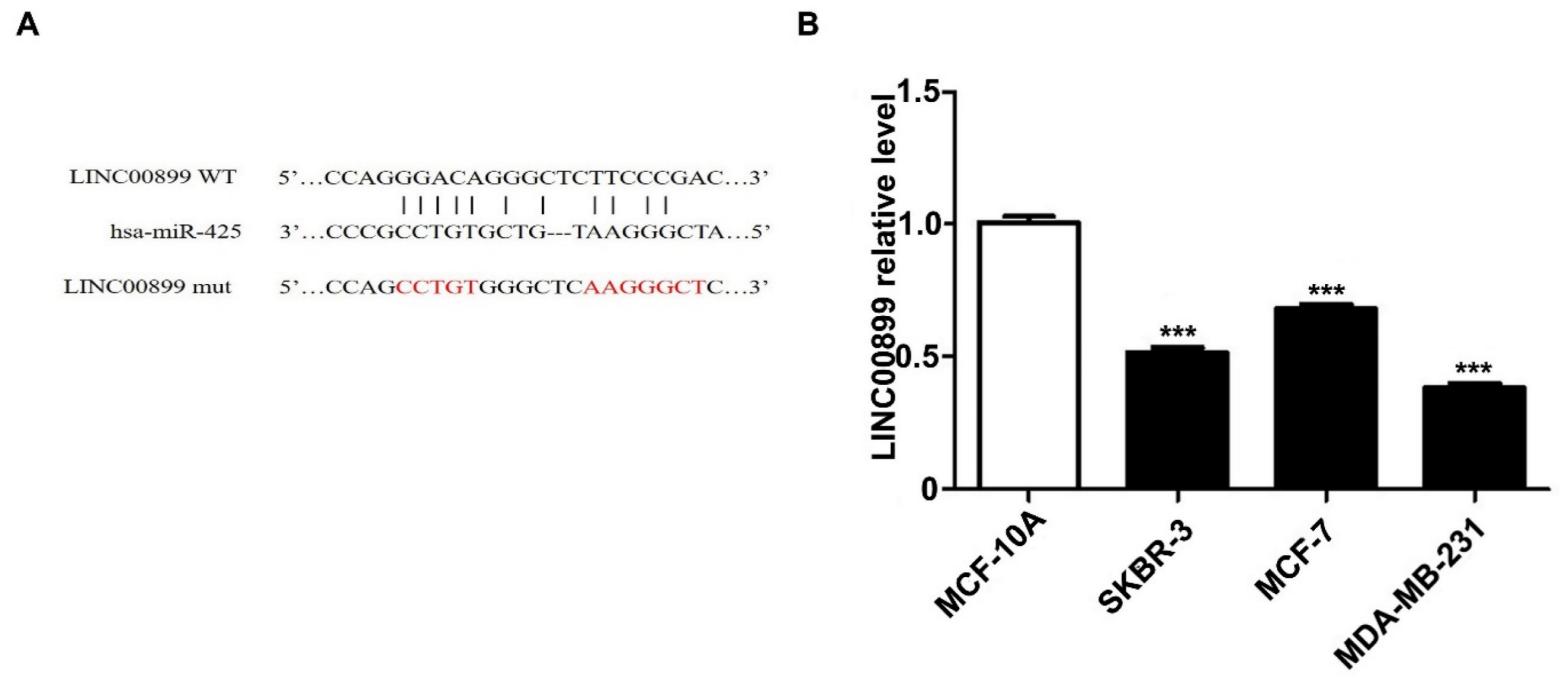
miR-425 is a target of LINC00899 in triple-negative breast cancer. (A) The predicted miR-425 binding site in the LINC00899 sequence. (B) MDA-MB-231 cells had the lowest expression level of LINC00899. ^***^P<0.005. miR, microRNA.

To explore the potential mechanism of LINC00899 in the progression of TNBC, miRcode and RNA22 were used to predict miRNAs that may interact with LINC00899. Of the 34 miRNAs differentially upregulated by the MD-MB-231 TNBC cell line compared with the HBL-10 breast epithelial cell line as determined by miRNA microarray analysis in a previous study (18) miR-425 was found to be a potentially notable target of LINC00899. Fig. 1A shows the predicted miR-425 binding site [wild type (wt) or mutant type (mut)] in the LINC00899 sequence. Zhou *et al* (5) demonstrated that miR-425 inhibited the luciferase activity of the LINC00899-wt reporter vector but hardly affected the LINC00899-mut reporter vector7. To investigate in which type of breast cancer cells LINC00899 has a notable biological role, RT-qPCR was used to detect the relative expression levels of LINC00899 in the MDA-MB-231 (triple-negative-type), MCF-7 (luminal-type), SKBR3 (HER2-type) and MCF-10A (normal human breast epithelial cells) cell lines. The results showed that LINC00899 expression was the lowest in MDA-MB-231 cells and highest in MCF-10A cells (Fig. 1B); therefore, the TNBC cell line was chosen as the study subject.

### 3.2 LINC00899 inhibits the proliferation and migration of MDA-MB-231 cells, and miR-425 reverses the tumor-inhibitory effect of LINC00899

**Figure 2:**
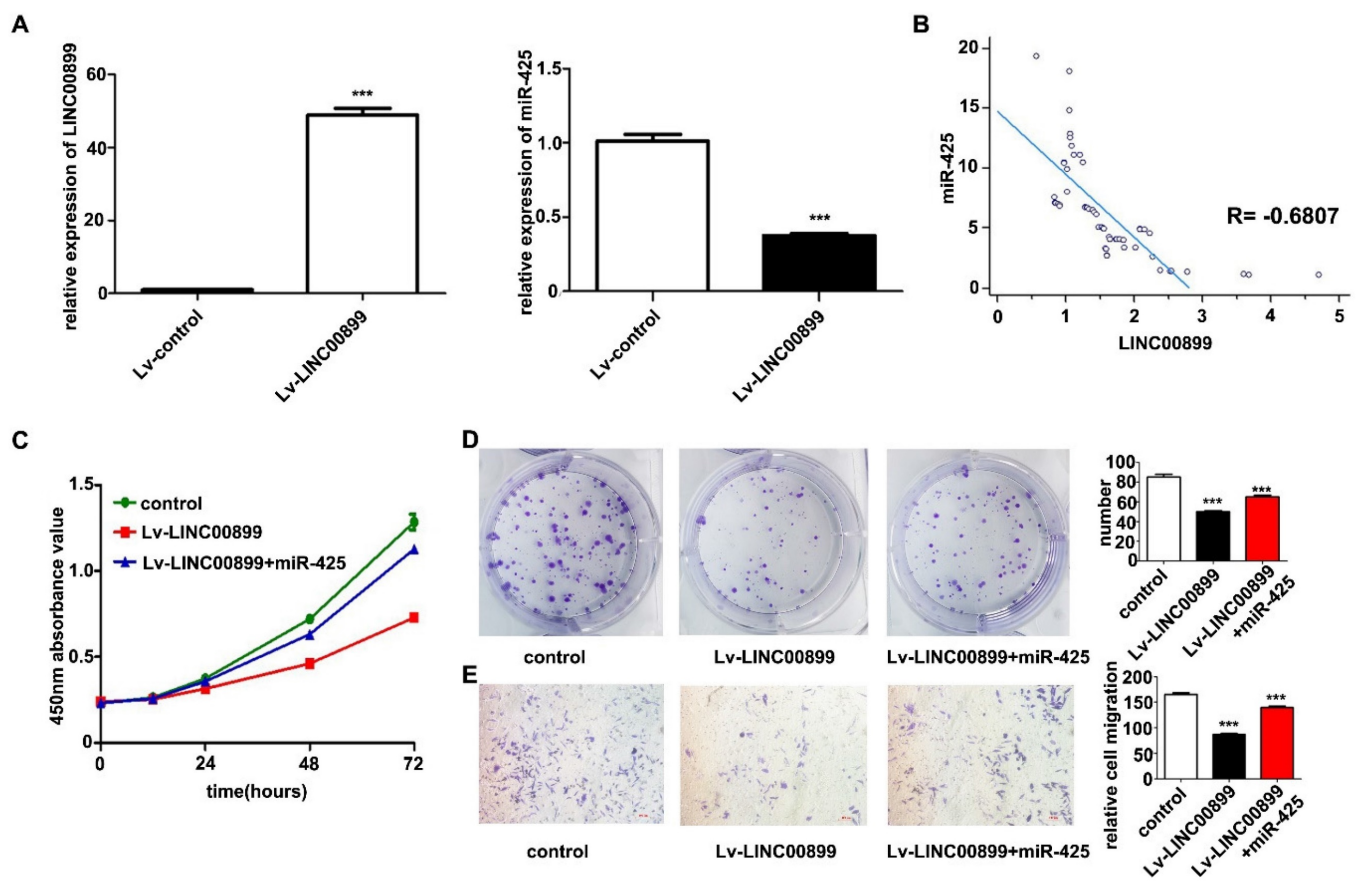
LINC00899 inhibits the proliferation of MDA-MB-231 cells and migration, and miR-425 can reverse the tumor-inhibitory effect of LINC00899. (A) Reverse transcription-quantitative polymerase chain reaction was used to detect the relative expression of miR-425 in each group. (B) LINC00899 was negatively correlated with the levels of miR-425 in plasma exosomes. (C) Cell Counting Kit-8 assay was used to detect cell viability. (D) Colony formation assay was used to detect proliferation (NC vs. lv-LINC00899, P=0.0004; NC vs. lv-LINC00899 + miR-425, P=0.0040; lv-LINC00899 vs. lv-LINC00899 + miR-425, P=0.0020). (E) Transwell assay was used to detect the migration ability (NC vs. lv-LINC00899, P<0.001; NC vs. lv-LINC00899 + miR-425, P=0.0042; lv-LINC00899 vs. lv-LINC00899 +miR-425, P=0.0001). ^***^P<0.005. lv, lentiviral; miR, microRNA; NC, negative control.

A LINC00899 overexpression vector or empty vector (negative control) was transfected into MDA-MB-231 cells for biological function validation. RT-qPCR was conducted to confirm that LINC00899 expression in the transfected MDA-MB-231 cells was significantly increased, while the expression of miR-425 was significantly decreased (Fig. 2A). In the plasma exosomes from patients with TNBC, LINC00899 expression was negatively correlated with the expression of miR-425 (r=-0.6807, P<0.001; Fig. 2B). The results of the CCK-8 assay showed that high LINC00899 expression decreased the viability of MDA-MB-231 cells (Fig. 2C), which was consistent with the colony formation assay results (Fig. 2D); furthermore, according to the results of the Transwell assay, high expression of LINC00899 significantly decreased the migration of MDA-MB-231 cells (Fig. 2E). Next, a miR-425 mimic was transfected into MDA-MB-231 cells overexpressing LINC00899. The results of the subsequent functional assays demonstrated that transfection with the miR-425 mimic reversed the LINC00899-mediated inhibition of the proliferation and migration of TNBC cells (Fig. 2C-2E). These results suggested that LINC00899 may target and regulate miR-425 in TNBC and that overexpression of miR-425 reversed the inhibitory effect of LINC00899 on TNBC.

### 3.3 miR-425 targets the regulation of PTEN

**Figure 3:**
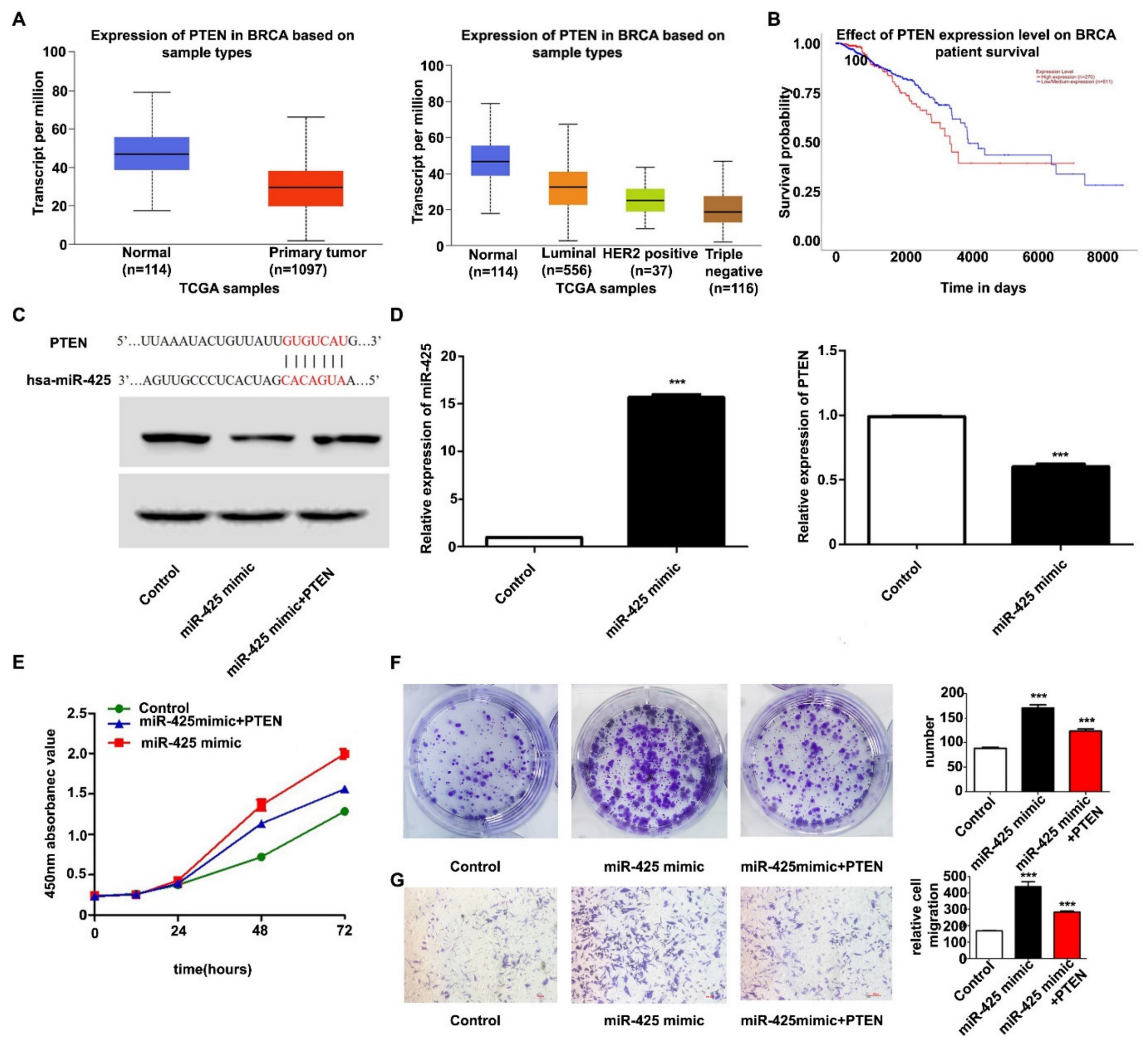
miR-425 promotes cell proliferation and migration by targeting PTEN. (A) TCGA database analysis to detect the relative expression of PTEN in breast cancer. (B) TCGA database analysis to determine whether PTEN can predict patient outcomes in breast cancer. (C) The predicted PTEN binding site in the miR-425 sequence. (D) Reverse transcription-quantitative polymerase chain reaction was used to detect the relative expression of miR-425 and PTEN in each group and western blotting was used to detect the relative protein expression of PTEN. (E) Cell Counting Kit-8 assay was used to detect cell viability. (F) Colony formation assay was used to detect proliferation (NC vs. miR-425 mimic, P<0.0001; NC vs. miR-425 mimic + PTEN, P=0.0018; miR-425 mimic vs. miR-425 mimic + PTEN, P=0.0035); (G) Transwell assay was used to detect the migration ability (NC vs. miR-425 mimic, P=0.0010; NC vs. miR-425 mimic + PTEN, P<0.0001; miR-425 mimic vs. miR-425 mimic + PTEN, P=0.0081). ^***^P<0.005. miR, microRNA; NC, negative control; PTEN, phosphatase and tensin homolog; TCGA, The Cancer Genome Atlas.

Candidate miR-425 target genes were searched for using publicly available databases (TargetScan; http://www.targetscan.org), which showed that miR-425 had 286 target genes. According to previous research and The Cancer Genome Atlas database, PTEN was poorly expressed in breast cancer and predicted patient outcomes (Fig. 3A and B). PTEN was therefore chosen as the downstream target gene of miR-425 for further study. Fig. 3C shows the predicted PTEN binding site in the miR-425 sequence. RT-qPCR and western blotting further confirmed that overexpression of miR-425 reduced the mRNA and protein levels of PTEN in MDA-MB-231 cells (Fig. 3D). These results suggested that miR-425 negatively regulated PTEN expression.

### 3.4 miR-425 promotes cell proliferation and migration mainly by targeting PTEN

It was next investigated whether PTEN, as a target of miR-425, has a role in regulating the proliferation and migration of TNBC cells. To this end, the control group or MDA-MB-231 cells overexpressing miR-425 were transfected with PTEN overexpression vector. The results of the subsequent functional experiments demonstrated that the overexpression of PTEN significantly attenuated miR-425-induced MDA-MB-231 cell proliferation and migration (Fig. 3E-G), indicating that miR-425 at least partially modulated the PTEN-induced proliferation and migration of MDA-MB-231 cells. These results suggested that PTEN may be a key target for miR-425 in regulating TNBC cell development.

### 3.5 Plasma exosomes from patients with TNBC contain low levels of LINC00899 and high levels of miR-425

**Figure 4:**
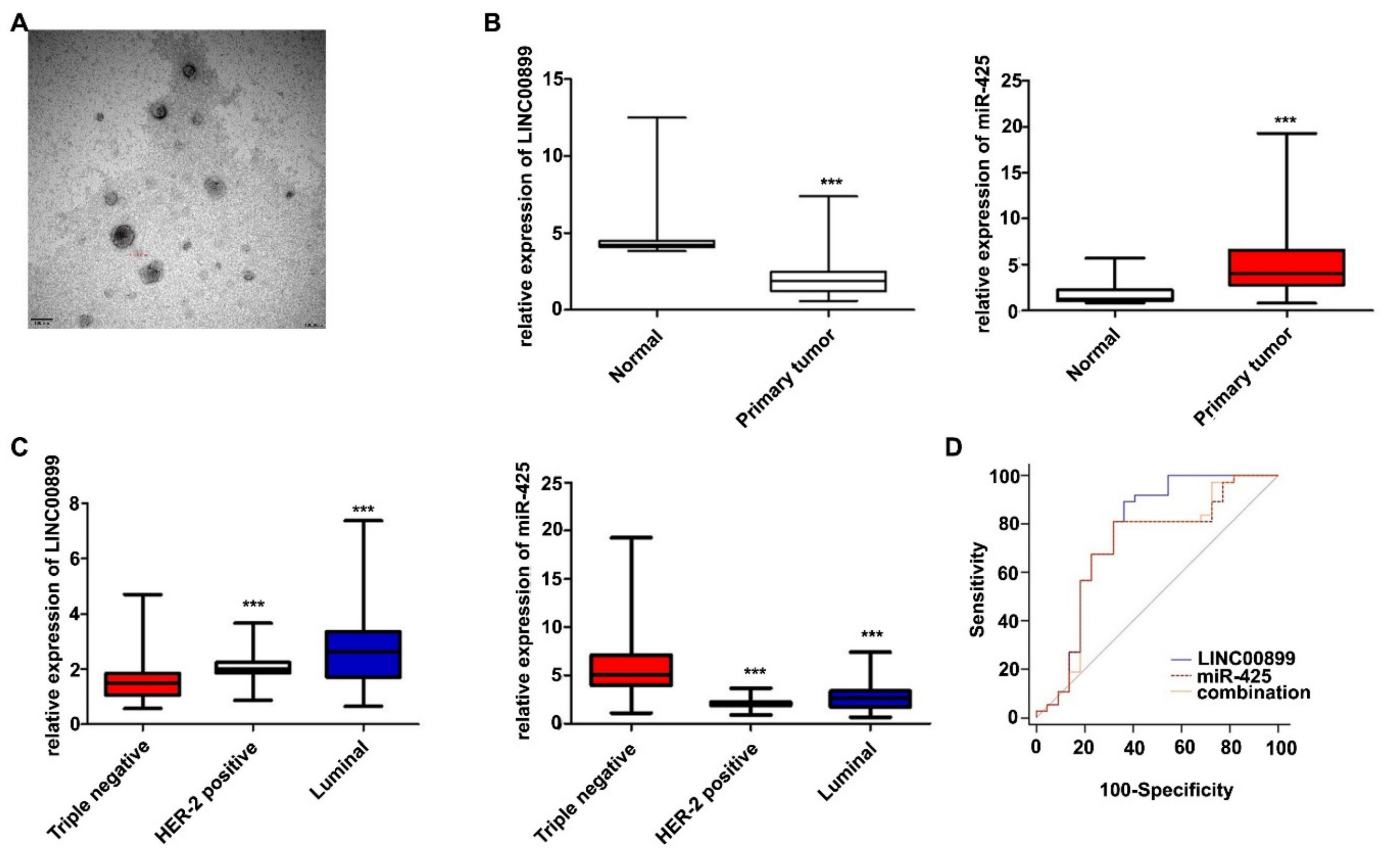
Exosomal LINC00899 and miR-425 can be used as biomarkers for the prognosis of patients with TNBC. (A) Plasma exosome electron microscopy images. (B) Differences in the relative expression of LINC00899 and miR-425 in patients with breast cancer and healthy women (^***^P<0.001). (C) Differences in the relative expression of LINC00899 and miR-425 in different subgroups of patients with breast cancer and healthy women (LINC00899: Triple negative vs. HER2^+^, P=0.0012; triple negative vs. luminal, P<0.001; luminal vs. HER2^+^, P=0.0692; miR-425: Triple negative vs. HER2^+^, P<0.001; triple negative vs. luminal, P<0.001; luminal vs. HER2^+^ P=0.0690). (D) Receiver operating characteristic curve analysis to assess the ability of the factors to predict the pathological outcome of patients with TNBC receiving neoadjuvant therapy (LINC00899, AUC=0.765; miR-425, AUC=0.706; LINC00899 + miR-425, AUC=0.708). AUC, area under the curve; miR, microRNA; TNBC, triple-negative breast cancer.

To further validate the effects of LINC00899 and miR-425 in patients with TNBC, plasma exosomes were collected from 119 patients with breast cancer treated with neoadjuvant chemotherapy (59 patients with TNBC, 32 with HER2^+^-type and 28 with luminal-type) and 20 healthy women. The TEM results showed that the extracted exosomes were between 80-110 nm in diameter (Fig. 4A). The RT-qPCR results showed that the LINC00899 levels in plasma exosomes from patients with breast cancer were lower than that in healthy women, while the miR-425 levels were higher than that in healthy women (Fig. 4B). The results of the breast cancer subgroup analysis showed that the LINC00899 levels were the lowest in the exosomes from patients with TNBC and the highest in patients with luminal breast cancer, while the miR-425 levels followed the opposite trend (Fig. 4C).

### 3.6 Exosomal LINC00899 and miR-425 can be used as prognostic biomarkers for patients with TNBC

Further analysis of the clinicopathological data of patients with TNBC showed that the LINC00899 levels were closely associated with the Ki-67 index, tumor size and lymph node metastasis, while there was no significant association with patient age, androgen receptor expression or vascular cancer thrombus (Table 2). According to the postoperative pathological results, the patients were divided into a pathological complete response group (no cancer residue in the breast or only a small amount of *in situ* ductal carcinoma residue and axillary lymph nodes without cancer residue) and a non-pathological complete response group. The ROC curve results showed that LINC00899 had a higher predictive value than miR-425 and the combination of LINC00899 and miR-425 for the pathological outcome of patients with TNBC receiving neoadjuvant treatment [LINC00899, area under the curve (AUC)=0.765; miR-425, AUC=0.706; combination, AUC=0.708; Fig. 4D].

## 4. Discussion

A number of studies have reported that lncRNAs are involved in various biological processes in breast cancer 20,21. Zhou *et al* 22 showed that TGFB2-AS1 inhibits the progression of TNBC through interaction with SMARCA4 and modulating SMARCA4. Zhang *et al* 23 found that the expression of lncRNA AFAP1-AS1 in TNBC tissue is significantly higher than that in normal tissue and other breast cancer subtypes, and that Sp1 is upregulated through sponging by miR-2110, thereby promoting the proliferation, migration and invasion of TNBC cells. The results of a study by Wang *et al* 24 indicated that linc-ZNF469-3 promotes lung metastasis of TNBC by regulating the miR-574-5p/zinc finger E-box binding homeobox 1 axis, which could be used as a potential biomarker to evaluate the prognosis of patients with TNBC. In addition, Zhou *et al* 25 showed that LINC00899 can significantly suppress the migration, invasion and proliferation of breast cancer cells by enhancing the expression of DICER1. However, to the best of our knowledge, the molecular mechanism by which LINC00899 regulates PTEN in TNBC has not yet been reported. In the present study, it was found that LINC00899 expression was associated with the Ki-67 index, tumor size and the presence or absence of lymph node metastasis, which could be used to evaluate the efficacy of neoadjuvant therapy in patients with TNBC. It was also found that overexpression of LINC00899 reduced the expression of miR-425/PTEN and inhibited the proliferation and migration of TNBC cells.

PTEN is a negative regulator of intracellular phosphatidylinositol triphosphate levels, and is the main negative regulator of the PI3K/AKT/mTOR signal transduction pathway, which is typically downregulated in breast cancer 26,27. The lack of specificity, sensitivity and inter-laboratory standardization in the determination of PTEN has led to controversy over its role as a prognostic and predictive biomarker for patients with breast cancer 28. Lower expression of PTEN in breast cancer tissue indicates poor prognosis in patients with TNBC 29. The HMG-box transcription factor 1/TIMP metallopeptidase inhibitor 3/PTEN axis inhibits the tumorigenesis of breast cancer and promotes the sensitivity of breast cancer to radiotherapy and hormone therapy 30. In addition, the miR-29a/PTEN/AKT axis, as an estrogen receptor α downstream signal transduction pathway, can control the progression and metastasis of breast cancer 31. The results of the present study suggested that high PTEN expression significantly attenuated miR-425-induced MDA-MB-231 cell proliferation and migration, indicating that miR-425 induced MDA-MB-231 cell proliferation at least partially by downregulating PTEN. These results suggested that PTEN may be a key target for miR-425 in regulating TNBC cell development.

In summary, the results of the present study demonstrated that LINC00899 was downregulated in TNBC tissues and predicted patient prognosis, and it was verified that the LINC00899/miR-425/PTEN axis regulated TNBC cell proliferation and migration. In conclusion, the results of the present study not only elucidated the underlying mechanism by which LINC00899 regulates TNBC progression but also suggested that the LINC00899/miR-425/PTEN axis may be a potential therapeutic target for TNBC.

## Figures and Tables

**Table 1:** Primer sequences used for quantitative reverse transcription polymerase chain reaction

	Forward and reverse primer sequences
*LINC00899*	Forward: 5'- CCCAACAGGAAGGTCTGGT-3'
	Reverse: 5'- TCAGTGCTGGGTCATTCTTG -3'
*MicroRNA-425*	Forward: 5'-TGCGGAATG ACACGATCACTCCCG-3'
	Reverse: 5'-CCAGTGCAGGGTCCGAGGT-3'
U6	Forward: 5'-CTCGCTTCGGCAGCACATATACT-3'
	Reverse: 5'-ACGCTTCACGAATTTGCGTGTC-3'
*PTEN*	Forward: 5'-TGTAAAGCTGGAAAGGGACGA-3'
	Reverse: 5'-GGAATAGTTACTCCCTTTTTGTCTC-3'
*GAPDH*	Forward: 5'-GGAGCGAGATCCCTCCAAAAT-3'
	Reverse: 5'-GGCTGTTGTCATACTTCTCATGG-3'

**Table 2:** Relationship between exosomal LINC00899 expression and clinicopathological features in patients with triple-negative breast cancer

Clinicopathologic features		Case (n)	LINC00899 relative expression		P
			High (n)	Low (n)	
Age	>50	32	17	15	0.7961
	<50	27	13	14	
Tumor size (cm)	2-5	48	29	19	0.0025
	>5	11	1	10	
lymph node	+	20	4	16	0.0009
	-	39	26	13	
Ki-67	≥30	49	21	28	0.0122
	<30	10	9	1	
AR	+	23	12	11	1.0000
	-	36	18	18	
Vascular cancer thrombus	+	9	4	5	0.7306
	-	50	26	24	

n: number AR: androgen receptor The P-value is calculated through Fischer test
